# Cocaine in Labor

**Published:** 1887-01

**Authors:** 


Cocaine in Labor.—Doleris applied a four per cent. solution or ointment to the vagina and external genitals, when the labor pains began. The amount of cocaine used was about two grains in each case. In this manner delivery was rendered painless in thirteen out of fifteen primiparæ. Fischel's results were less striking, but he employed weaker solutions. These observations show that the seat of the labor pains is not in the uterus, but in the dilating cervix and vagina.—*Med. Age.*

